# Performance effects of internal pre- and per-cooling across different exercise and environmental conditions: A systematic review

**DOI:** 10.3389/fnut.2022.959516

**Published:** 2022-10-14

**Authors:** Maria Roriz, Pedro Brito, Filipe J. Teixeira, João Brito, Vitor Hugo Teixeira

**Affiliations:** ^1^Faculty of Nutrition and Food Sciences, University of Porto (FCNAUP), Porto, Portugal; ^2^Futebol Clube do Porto, Porto, Portugal; ^3^Research Center in Sports Sciences, Health Sciences and Human Development, CIDESD, University of Maia, ISMAI, Maia, Portugal; ^4^Interdisciplinary Center for the Study of Human Performance (CIPER), Faculdade de Motricidade Humana, Universidade de Lisboa, Cruz-Quebrada, Portugal; ^5^Atlântica, Instituto Universitário, Fábrica da Pólvora de Barcarena, Barcarena, Portugal; ^6^Bettery Lifelab, Bettery S.A., Lisbon, Portugal; ^7^Portugal Football School, Portuguese Football Federation, Oeiras, Portugal; ^8^Research Centre in Physical Activity, Health and Leisure (CIAFEL), Faculty of Sports, University of Porto (FADEUP), Porto, Portugal; ^9^Laboratory for Integrative and Translational Research in Population Health (ITR), Porto, Portugal

**Keywords:** exercise, heat, internal cooling, nutrition, sports performance

## Abstract

Exercise in a hot and humid environment may endanger athlete’s health and affect physical performance. This systematic review aimed to examine whether internal administration of ice, cold beverages or menthol solutions may be beneficial for physical performance when exercising in different environmental conditions and sports backgrounds. A systematic search was performed in PubMed, Web of Science, Scopus and SPORTDiscus databases, from inception to April 2022, to identify studies meeting the following inclusion criteria: healthy male and female physically active individuals or athletes (aged ≥18 years); an intervention consisting in the internal administration (i.e., ingestion or mouth rinse) of ice slush, ice slurry or crushed ice and/or cold beverages and/or menthol solutions before and/or during exercise; a randomized crossover design with a control or placebo condition; the report of at least one physical performance outcome; and to be written in English. Our search retrieved 2,714 articles in total; after selection, 43 studies were considered, including 472 participants, 408 men and 64 women, aged 18-42 years, with a VO_2max_ ranging from 46.2 to 67.2 mL⋅kg^–1^⋅min^–1^. Average ambient temperature and relative humidity during the exercise tasks were 32.4 ± 3.5°C (ranging from 22°C to 38°C) and 50.8 ± 13.4% (varying from 20.0% to 80.0%), respectively. Across the 43 studies, 7 exclusively included a menthol solution mouth rinse, 30 exclusively involved ice slurry/ice slush/crushed ice/cold beverages intake, and 6 examined both the effect of thermal and non-thermal internal techniques in the same protocol. Rinsing a menthol solution (0.01%) improved physical performance during continuous endurance exercise in the heat. Conversely, the ingestion of ice or cold beverages did not seem to consistently increase performance, being more likely to improve performance in continuous endurance trials, especially when consumed during exercises. Co-administration of menthol with or within ice beverages seems to exert a synergistic effect by improving physical performance. Even in environmental conditions that are not extreme, internal cooling strategies may have an ergogenic effect. Further studies exploring both intermittent and outdoor exercise protocols, involving elite male and female athletes and performed under not extreme environmental conditions are warranted.

**Systematic review registration:** [https://www.crd.york.ac.uk/prospero/display_record.php?ID=CRD42021268197], identifier [CRD42021268197].

## Introduction

Prolonged exercise in hot and humid environments is challenging for health and athletic performance, mainly during endurance and team-sport activities (1, 2). Actually, when environmental conditions are extreme, temperature regulation mechanisms may be disrupted and may not compensate the elevation of core body temperature (3). However, the mechanisms underpinning fatigability and reduced performance during exercise in hot environments are likely to be multifactorial.

Overall, body temperature is regulated by both physiologic and behavioral mechanisms (4, 5). Briefly, physiological temperature regulation operates through responses that are independent of conscious voluntary behavior, and include cutaneous vasodilatation (increasing skin blood flow) and increments pertaining sweat rate (3, 5). On the other hand, behavioral temperature regulation occurs through conscious behavior changes that influence heat accumulation, and include modification of the activity levels, clothing changes and seeking of shade or shelter (4). During exercise under heat stress, the rate of heat production is greater than the rate of heat loss and may lead to hyperthermia (6). Also, a greater cardiovascular distress should be expected because the main challenge is to provide sufficient cardiac output to adequately perfuse skeletal muscles to support metabolism, while simultaneously perfusing the skin to support heat loss (7, 8). Thus, hyperthermia is known to alter cardiovascular function, reducing physical and athletic performance (9).

Several cooling strategies have been tested and used with the primary goal of reducing central temperature and thermal sensation, and further delaying the onset of fatigue (10, 11). Concomitantly, cooling methods can be either applied prior to or during exercise. As a pre-cooling strategy, cooling methods should aim to increase the margin for metabolic heat production and heat increase, while as a per-cooling strategy, the aim is to attenuate the rise of exercise-induced central temperature and to cool the body when already under heat stress (12, 13). Though, the combination of pre-cooling and per-cooling techniques seems to be more effective in improving exercise performance in the heat than any method applied individually (10).

Similarly, diverse external and internal thermal cooling techniques are often used to reduce the detrimental effects of heat stress. Notably, external methods (e.g., using a cooling vest, ice pack, cold-water immersion) have proved to be effective in decreasing core, muscle and skin temperatures, and improving physical performance (10), whether internal methods (e.g., ice slurry, crushed ice, cold beverage ingestion) have shown to decrease brain temperature and to improve thermal perception via stimulation of thermoreceptors located within oral and abdominal regions (10, 14). Recently, the research on non-thermal cooling methods, either external or internal, has grown, with particular attention to L-menthol, due to its properties in relieving the thermal strain associated with exercise in the heat (15, 16). L-menthol can be applied topically (creams, gels, or sprays) or internally, via mouth rinse or ingestion of menthol flavored solutions (17).

The effects of external cooling methods on sports performance are well documented (18, 19). However, no systematic review has exclusively focused on the effects of internal thermal and non-thermal cooling strategies on physical performance, specifically in different types of exercise backgrounds. This is of utter importance, because more and more sport events worldwide will occur under adverse environmental conditions. Also, a better understanding of such strategies may provide practical and cost-effective insights on how to improve athletic performance in the heat. Therefore, the purpose of the current systematic review is to summarize the existing scientific literature regarding the role of internal administration of cooling methods in physical performance, applied either before and/or during exercise, in healthy physically active male and female individuals or athletes, in different environmental and exercise contexts.

## Materials and methods

This systematic review followed the Preferred Reporting Items for Systematic Reviews and Meta-Analyses (PRISMA) guidelines (20). The protocol was previously registered at the International Prospective Register of Systematic Reviews (PROSPERO) under registration number CRD42021268197.

### Search strategy

All studies were identified through a search on four electronic databases (PubMed, Web of Science, Scopus and SPORTDiscus) from their inception until the 4th of April 2022. Reference lists of the included articles were also searched for additional references to be included in case they would fulfill all inclusion criteria. There was no limit on the status, language, and dates of publication. Twenty-two terms and keywords (“exercise,” “sports performance,” “athletic performance,” “sports,” “physical activity,” “athletes,” “pre-cooling,” “cooling,” “per-cooling,” “mid-cooling,” “body temperature,” “heat mitigation,” “ice-slurry,” “ice-slushy,” “ice-slush,” “beverage,” “drinking,” “cold fluid,” “cold water,” “menthol,” “mint,” “peppermint”) were combined by Boolean logic operators (AND) and (OR) (Supplementary Appendix A).

### Eligibility criteria

The specified eligibility criteria for the systematic review research followed the PICOS model eligibility criteria, which consider the factors of population (P), intervention (I), comparators (C), and outcomes (O), along with study design (S) (21).

#### Inclusion criteria

To be considered for analysis, the studies must have: (1) involved male or female healthy physically active or athletes (age ≥18 years) subjects; (2) an intervention that includes the internal administration (ingestion or mouth rinse) of ice slush, ice slurry or crushed ice and/or cold beverage and/or menthol solution before and/or during exercise; (3) reported at least one physical performance outcome (i.e., time to exhaustion, time-trials, distance covered, strength and power, exercise capacity, sprint velocity, etc.); (4) a randomized controlled crossover design, with a control or placebo group; and (5) been written in English.

Authors opted to only include trials with a crossover design because such research design has strong advantages over parallel group trials, particularly in this research area (22). Physical performance is highly variable between participants, as it is conditioned by individual factors. So, a crossover design, where the interventions under investigation are evaluated within the same patients, eliminating between-subject variability, best serves the purpose of our review.

#### Exclusion criteria

Studies were excluded if they: (1) were conducted in participants with injury or illness; (2) were secondary design studies (such as meta-analyses, systematic reviews and narrative reviews), animal studies, articles with no full-text available, opinion pieces, commentaries, editorials, letters, theses, meeting abstracts or “gray literature” in general; (3) included exclusively external cooling or did not report an exclusive effect of the internal cooling; and (4) evaluated only outcome measures based on non-physical performance parameters (e.g., physiological markers).

### Study selection

Following the initial search in each database, all references located were imported into EndNote X20 (Clarivate Analytics, London, United Kingdom). Two independent authors (MR and PB) selected the eligible articles, based on the title and abstract, and removed duplicated articles. The full text of references identified from the previous process was reviewed and assessed independently by two reviewers (MR and PB) using the inclusion and exclusion criteria. Disagreements were solved by consensus between the two researchers (MR and PB) after discussion. Unresolved discrepancies were settled by a third reviewer (VHT).

### Data extraction

In the set of included studies, data extraction was completed independently by two authors (MR and PB) to an Excel spreadsheet to collect information for descriptive purposes. The last author of this review (VHT) supervised the process. The following data were extracted and presented in Table 1 as follows: authors and year of publication, ambient conditions (temperature, relative humidity, and wind speed), sample size, characteristics of the participants (sex, age, and training status), study design, exercise protocol, cooling timing, cooling technique, and physical performance outcomes.

**TABLE 1:** Summary of the included studies involving an intermittent exercise protocol.

Study	Ambient conditions	Participant characteristics	Design	Exercise protocol	Cooling timing	Cooling technique	Performance outcomes
**Menthol**							
Best et al. (106)	22 ± 1°C	Recreational male athletes (n = 10). Age 24.6 ± 3.9 y. Recreational female athletes (*n* = 9). Age 20.2 ± 1.0 y.	Crossover, counterbalanced, randomized	3x Isometric Mid-Thigh Pull + 3x Vertical Jump + 3 × 6 s Peak Power (cycle ergometer)	Per-cooling	25 mL menthol solution mouth rinse (0.1%, 10 s) 60 s before each exercise effort	↓1.4% IMTP ↑0.9% VJH ↓1.5% PP (p = 1.000)
Gibson et al. (103)	35 ± 0.2°C 40 ± 0.5% RH	Non-heat-acclimated trained team sports male (*n* = 11) and female (*n* = 3). Age 24 ± 3 y. VO_2_max 46.2 ± 12.9 mL⋅kg ^–1^⋅min^–1^	Crossover, randomised	CISP protocol	Per-cooling	25 mL L-menthol solution mouth rinse (0.01%, 5 s, ∼40°C) every 10 min	↔ PP, MP, WD (p > 0.05)
**Ice/Cold beverages**							
Aldous et al. (119)	30.7 ± 0.3°C 50.9 ± 4.2% RH	University-level male football players (*n* = 8). Age 22 ± 3 y. VO_2_max 56 ± 9 mL⋅kg^–1^⋅min^–1^	Crossover, counterbalanced, randomized	2 × 45 min iSPT	Pre-cooling Per-cooling	7.5 g kg^–1^ ice slurry ingestion (-1°C) 30 min before exercise + 3.75 g kg^–1^ ice slurry ingestion (-1°C) during 15 min half-time	↔ TD, HSD, VRD
Beaven et al. (134)	25°C 60% RH	Professional rugby sevens male athletes (n = 12). Age 21.5 ± 1.3 y	Crossover, counterbalanced, randomised	5 × 40 min maximal running sprint every 30 s	Pre-cooling	500 mL of non-calorific ice slushy ingestion 15 min before exercise	**↓3.2% RT (*p* = 0.0015)** ↑1.9% ST (p > 0.05)
Brade et al. (132)	35.2 ± 0.3°C 57.8 ± 1.2% RH	Male team sport players (n = 12). Age 21.8 ± 2.3 y	Crossover, counterbalanced, randomized	70 min sprint cycling (2 × 30 min halves + 10 min interval)	Pre-cooling Per-cooling	7.0 g kg^–1^ of ice slushy ingestion (0.6°C) 30 min before exercise + 2.1 g kg ^–1^ of ice slushy ingestion (0.6°C) during half-time	↔ PPO ↔ MPO ↔ PP ↔ MP ↔ W (p > 0.05)
Gerret et al. (122)	30.9 ± 0.9°C 41.1 ± 4.0% RH 1.3 m s^–1^ WS	Moderately to well-trained males (n = 12). Age 30.4 ± 3.4 y. VO_2_max 58.5 ± 8.1 mL⋅kg^ –1^⋅min^–1^	Crossover, randomized	2 × 31 min self-paced intermittent protocols	Pre-cooling	7.5 g kg^ –1^ ice slurry ingestion (0.14 ± 0.1°C) 30 min before exercise	↑1.5% TD (p > 0.05)
Hue et al. (102)	Water temperature 29.5 ± 0.5°C	Ranked long-distance male (n = 5) and female (n = 4) swimmers. Age 23.4 ± 3.3 y	Crossover, randomized	10 × 100 m swimming (5000 m)	Per-cooling	190 mL cold water ingestion (1.3°C) before and after each 1,000 m	↔ Swimming time (p > 0.05)
Lafata et al. (97)	WBGT = 15°C DBT = 24°C	Healthy, physically fit male (*n* = 52). Age 30.3 ± 5.4 y. VO_2_max 49.8 ± 6.3 mL⋅kg ^–1^⋅min^–1^	Crossover, randomized	60% 1RM bench press to fatigue + broad jump, + cycling TTE	Per-cooling	12 mL kg ^–1^ cold beverage ingestion (4°C) during the rest period between sets of exercises	↑0.7% TTE (*p* = 0.7035) ↑0.9% BJ (*p* = 0.465)
Naito et al. (93)	36.5 ± 0.5°C 50 ± 3% RH	Non-heat acclimated physically active males (*n* = 7). Age 31 ± 4 y	Crossover, randomized	Intermittent cycling protocol	Per-cooling	1.25 g kg^–1^ ice slurry ingestion (−1°C) during each break + 7.5 g kg^–1^ ice slurry ingestion (−1°C) during 10 min half-time	↔ MPO, PPO (*p* > 0.05) **↓ 4.2% WD (*p* < 0.05)**
Thomas et al. (127)	34.4 ± 1.4°C 36.3 ± 4.6% RH	Healthy trained male (*n* = 10). Age 30.5 ± 5.8 y. VO_2_max 56.2 ± 6.6 mL⋅kg ^–1^⋅min^–1^	Crossover, counterbalanced, randomized	46-min intermittent protocol	Pre-cooling	7.5 g kg^–1^ ice slurry ingestion (−0.5 ± 0.4°C) 30 min before exercise	↑4% TD (p > 0.05)
Zimmermann and Landers (99)	33.1 ± 0.1°C 60.3 ± 1.5% RH	Trained team sports female (*n* = 9). Age 21.0 ± 1.2 y	Crossover, randomized	72 min (2 × 36 min) intermittent sprint cycling	Pre-cooling	6.8 g kg^–1^ crushed ice ingestion (^–^0.5°C) 30 min before exercise	↔ PPO (*p* = 0.799) ↔ MPO (*p* = 0.989)

↔ no change, ↑ increase, ↓ decrease, BJ: broad jump, CISP cycling intermittent-sprint protocol, DBT: dry-bulb temperature, HSD: high-speed distance covered, IMTP: isometric mid-thigh pull, kJ: kilojoules, kg: kilograms, km: kilometers, m: meters, mL: milliliters, min: minutes, MEN: menthol, MP: mean power, MPO: mean power output, P: power, PO: power output, PP: peak power, PPO: peak power output, RH: relative humidity, RT: rate of fatigue, s: seconds, ST: sprint time; T: time, TD: total distance covered, VJH: vertical jump height, VO2max: maximal oxygen uptake, VRD: variable run distance covered, W: work, WBGT: wet-bulb globe temperature, WBT: wet bulb temperature, WD: total work done, WS: wind speed and y: years. p: significance level. Bold represents a significant p-value at a confidence level of 95% (p < 0.05).

### Risk of bias assessment

Risk of bias was assessed according to Cochrane Collaboration guidelines (23) (Risk of Bias Tool V.2.) at study level, using Review Manager 5.4, encompassing seven domains: sequence generation, allocation concealment, blinding of participants and outcome assessors, incomplete outcome data, selective outcome reporting and other bias. Each potential source of bias was graded as low, high, or unclear risk. The process was carried out by two of authors independently (MR and PB) that underwent a calibration exercise before performing the assessment of risk of bias. When the details of a study were unclear, the authors were contacted to provide further information/details. Conflicts were settled through discussion amongst the pair of reviewers or through consultation with a third reviewer (VHT).

## Results

### Literature search

**FIGURE 1:**
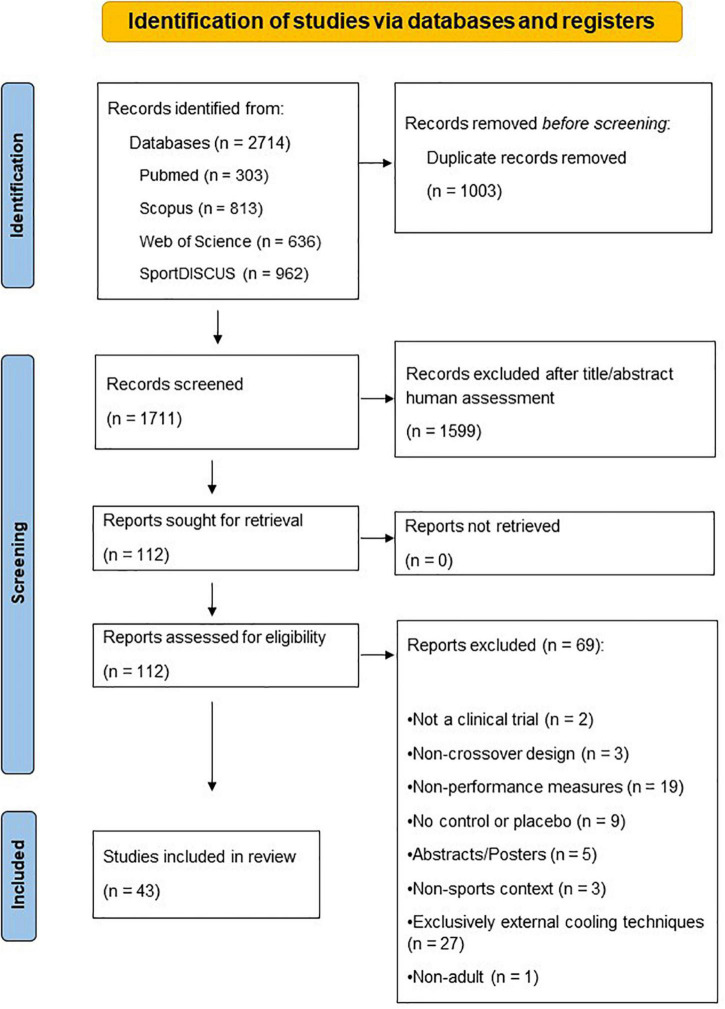
PRISMA flow diagram summarizing study selection for inclusion in systematic review.

The database search retrieved 2,714 articles. These were reduced to 1,711 after removal of duplicates (*n* = 1,003). Further screening by title and/or abstract analysis excluded 1,599 articles. The 112 studies left were assessed for eligibility via full text review, and references lists did not reveal any missing papers. The exclusion criteria determined a further removal of 69 articles. Two were review articles (24, 25), three had a non-crossover design (26–28), nineteen reported only physiological, cognitive or perceptual performance measures (29–47), nine had no control or placebo group (48–56), five were conference abstracts/posters (57–61), three were performed in clinical context (62–64), twenty-seven did not report an exclusive effect of the internal cooling (65–91) and one comprised underage participants (92). Forty-three articles fulfilled the eligibility criteria and data were extracted for qualitative analysis. Figure 1 details the study search, identification, and selection process using the PRISMA flow diagram.

### Characteristics of the included studies

#### Participants

Overall, 472 subjects (408 male and 64 female) from 43 studies were included in the qualitative analysis, with the number of participants ranging from 7 (93–96) to 52 (97). Thirty-four studies assessed only male participants, three only females (98–100), and six included a mixed-sex sample (101–106). The average age of the participants was 27.1 years (ranging between 18 (107) and 42 years (108)). The average VO_2_max of the participants was 56.0 mL⋅kg^–1^⋅min^–1^ (ranging from 46.2 (103) to 67.2 mL⋅kg^–1^⋅min^–1^ (109)). Seventeen studies included physically active participants (93, 94, 97, 101, 104, 105, 107, 110–118), twenty-one included recreational athletes (95, 96, 98–100, 106, 108, 119–131), and five involved professional athletes (102, 109, 132–135).

#### Exercise protocols and environmental conditions

The exercise protocols involved were divided as the following:

(A). Continuous endurance exercise (*n* = 32).

• Time-trials (*n* = 14) (94, 96, 98, 100, 104, 108, 109, 123–126, 129, 130, 133).
• Time to exhaustion (*n* = 17).
∘ at fixed first ventilatory threshold (VT1) (*n* = 2) (113, 114).
∘ at fixed rate of perceived exertion (RPE) (*n* = 3) (101, 110, 121).
∘ at fixed VO_2_peak or at maximal aerobic power output (W_max_) or at respiratory compensation point (*n* = 12) (94, 95, 107, 111, 112, 115–118, 120, 128, 131).
• Maximum power effort (*n* = 1) (105).

(B). Intermittent effort exercise (*n* = 11) (93, 97, 99, 102, 103, 106, 119, 122, 127, 132, 134).

Ambient temperature during the exercise tasks ranged from 22°C (106) to 38°C (131) and relative humidity varied between 20% (126) and 80% (123). Forty studies included indoor protocols and three were performed outdoor (102, 104, 130). Wet bulb globe temperature (WBGT) was calculated from each of the included studies (136), with the exception of the studies where WBGT was recorded and reported and other two studies where WBGT could not be calculated (one carried out in water (102) and another study that did not indicate the value of relative humidity (106)). A WBGT ≥28°C was used as a cut-off point to group studies concerning environmental conditions (137). Thirty-four studies were performed under a WBGT ≥28°C (53, 93–96, 98–101, 103, 105, 107–113, 115, 116, 119, 121–123, 125–129, 131–133, 138) and seven studies under a WBGT <28°C (97, 104, 117, 118, 130, 134, 139).

#### Interventions

All the included studies analyzed the effect of pre-cooling and/or per-cooling with non-thermal (menthol solution) and/or thermal (ice or cold beverages) methods on physical performance, compared with non-cooling control or placebo.

Across all the 43 studies, seven exclusively included a menthol solution mouth rinse (100, 101, 103, 106, 110, 111, 117), thirty exclusively involved ice slurry/ice slush/crushed ice/cold beverages intake (61, 80, 93–99, 102, 104, 107, 109, 112–115, 118–127, 132–134), and six examined both the effect of thermal and non-thermal internal techniques in the same protocol (105, 108, 116, 128–130).

All studies that exclusively applied non-thermal methods had a per-cooling mode (100, 101, 103, 106, 110, 111, 117). Studies that involved thermal strategies encompassed the ingestion of ice or cold beverages both before and during exercise (107, 119, 120), only before exercise (61, 94, 96, 98, 99, 104, 114, 115, 121–125, 127, 134) or only during exercise (93, 95, 97, 102, 107, 109, 112, 113, 118, 126, 133). In the six studies where thermal and non-thermal strategies were both included, the techniques employed were the administration of cold water or menthol solution before exercise (105), ice slurry before exercise or menthol solution during exercise (129), ice slurry or menthol solution during exercise (116), ice or cold menthol flavored beverages before and during exercise (108), and crushed ice before exercise and menthol solution during exercise (128).

### Risk of bias assessment

**FIGURE 2:**
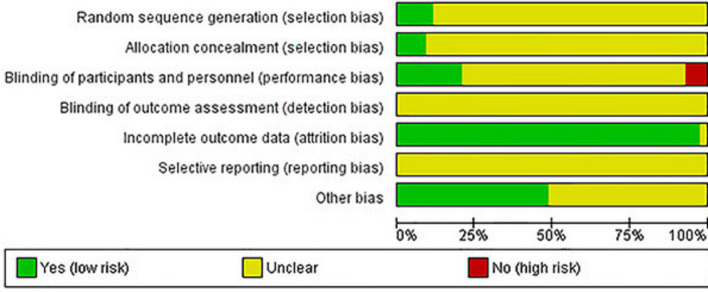
Risk of bias graph: review authors’ judgments about each risk of bias item presented as percentages across all included studies.

**FIGURE 3:**
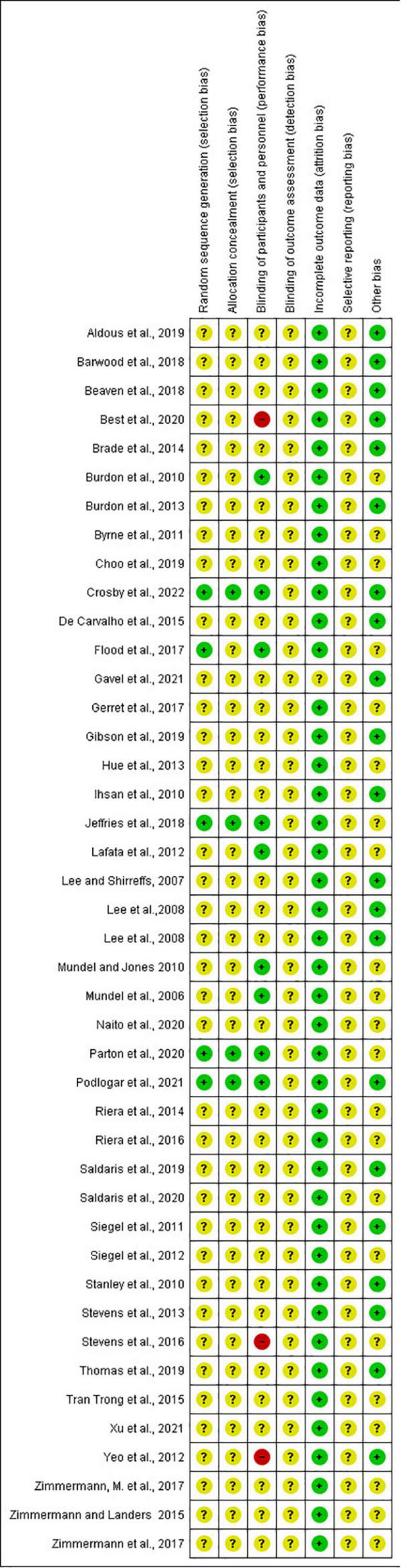
Risk of bias summary: review authors’ judgments about each risk of bias item for each included study.

The included studies generally had low or unclear risk of bias (Figures 2, 3). Only five studies had a low risk of sequence generation bias, because they reported information on the randomization procedure conducted to generate groups (101, 105, 110, 116, 117). Attempts to conceal allocation to an intervention or control group were only well reported on four studies (101, 105, 116, 117), with the others having an unclear risk of bias. Three studies (105, 110, 116) were single-blinded, two studies were performed in a double-blinded fashion (101, 117), and four studies (95, 97, 111, 112) reported having blinded the participants to the purpose of the intervention and thence. Four studies clearly reported no blinding of the participants to the purpose of the study (104, 106, 129, 132), which represents a high risk of bias. In each one of the included studies, it was not clear whether the blinding of outcome assessment occurred, so an unclear risk of bias in this category was considered. All studies were considered to have a low risk of bias for incomplete data and an unclear risk for selective reporting. Finally, regarding other bias, twenty-one studies did not report if beverages were distributed in a counterbalanced order, so a bias related to the distribution order was not clear (93–95, 97–99, 101, 102, 108, 110, 111, 114–116, 121–123, 128–131). A study did not set the amount of beverage to be consumed between groups (*ad libitum* fluid ingestion), meaning that it was not clear if the drinking rate may have influenced the outcomes (112).

### Results of the included studies

The summary of outcomes measures and main findings of the forty-three included studies are presented in Table 1 and Supplementary Table 1.

#### Menthol solution *vs.* non-cooling strategy

##### Intermittent exercise protocols

**FIGURE 4:**
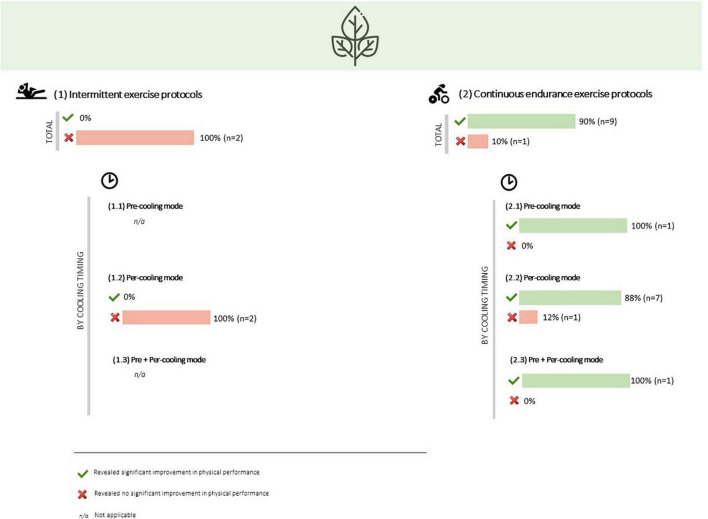
Overall results of oral menthol administration effect on physical performance, by type of exercise and cooling timing.

Mouth rinsing a menthol solution during an intermittent exercise protocol (3 x isometric mid-thigh pull + 3x vertical jump + 3 × 6 s of peak power on a cycle ergometer) showed no significant improvement in none of these performance parameters (106). Similarly, oral menthol administration during a Cycling Intermittent-Sprint Protocol (CISP), which consisted of 10-s rest, 5-s maximal sprint, and 105-s active recovery with the cycles repeated over 10 min (for 20 min), revealed no significant effect on peak power, mean power and total work done (103) (Table 1 and Figure 4).

##### Continuous endurance exercise protocols

In the only study where menthol was internally applied in a pre-cooling mode, specifically in the minute before a 3-min aerobic test exercise (an exercise protocol much shorter than the other ones), a significant improvement in relative power output was found (13%) (105).

Internal application of menthol solution during continuous exercise significantly enhanced endurance performance in seven out of eight studies. Power output (3.6-6.5%) and completion time (4-7%) were significantly improved at a 16 fixed-RPE cycling protocol (101, 110). Time to exhaustion was significant higher (6-8.6%) with menthol mouth rinsing during a 65 and 70% maximal aerobic power output cycling protocols (111, 116), as well as time to fatigue (34.4%) in a running protocol with an intensity of 100% of VO_2_peak (128). Time trial efforts also showed significant improvements while per-cooling with oral menthol (2.3-2.7%), namely in a 30-km (100) cycling and in a 5-km running protocols (129). However, one study did not find a significant improvement in time to exhaustion when mouth rinsing with a menthol-containing sports drink during a 105% of respiratory compensation point cycling protocol (117).

When oral menthol was administered before and during exercise, which only occurred in one study, completion time was significantly improved (5.3%) in a 20-km time trial cycling protocol (108) (Supplementary Table 1 and Figure 4).

##### Influence of environmental conditions

Overall, nine of the ten studies that involved the administration of oral menthol and were performed under a WBGT ≥28°C found significant improvements in physical performance (100, 101, 105, 108, 110, 111, 116, 128, 129). When stratifying the analysis by type of exercise, it is possible to observe that the only study that did not report significant improvements in physical performance in more adverse environmental conditions involved an intermittent exercise protocol (103).

**FIGURE 5:**
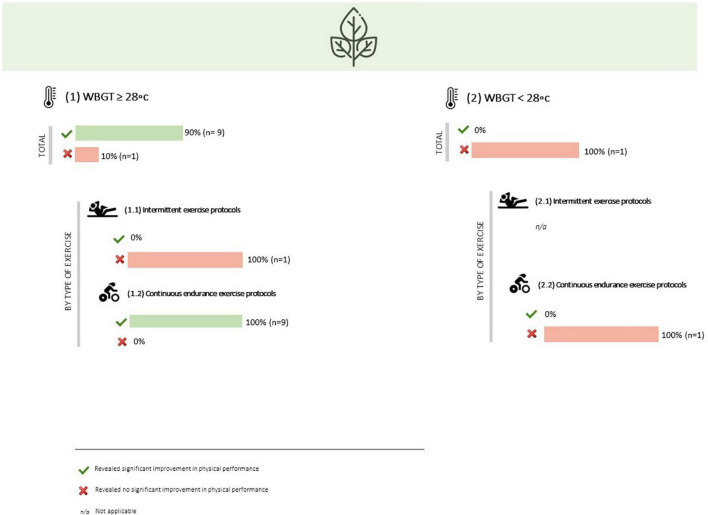
Overall results of oral menthol administration effect on physical performance, by environmental conditions and type of exercise.

On the other hand, the only study that involved menthol mouth rinsing and was completed under a WBGT <28°C did not report significant improvements in physical performance (117), and involved a continuous endurance exercise protocol, with a time to exhaustion at 105% of respiratory compensation point (Figure 5).

#### Ice or cold beverage *vs.* non-cooling strategy

##### Intermittent exercise protocols

In the four studies where ice or cold beverages were ingested before intermittent exercise, only one found significant improvements in physical performance, specifically on rate of fatigue (3.2%) in a 5 × 30-s of maximal running sprint protocol, even though sprint time was not significantly improved (134). Total distance in a 2 × 31-min (122) and in a 46-min (127) intermittent protocols on non-motorized treadmill was not enhanced by pre-cooling with ice slurry. No significant differences were found in peak power and mean power outputs in a 2 × 36-min intermittent sprint cycling protocol (99).

Across the three studies where ice or cold beverages were ingested during intermittent exercise, none showed a significant ergogenic effect, neither on a 10 × 100-m swimming protocol (101), or in a repeated sprint intermittent cycling protocol (92) or even in a mixed-tasks protocol with bench press to fatigue + broad jump + self-paced cycling time to exhaustion (96).

**FIGURE 6:**
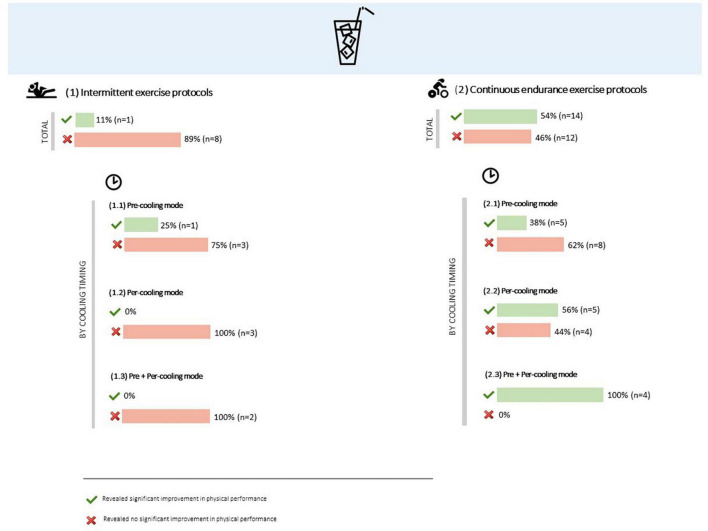
Overall results of ice or cold beverages administration effect on physical performance, by type of exercise and cooling timing.

In the set of the two studies where ice or cold beverages were administered before and during intermittent exercise, none reported significant differences in physical performance. Exercise protocols involved a 2 × 45-min iSPT (consisting of three identical 15-min intermittent exercise blocks) (119) and a 2 × 30-min sprint cycling exercise (132) (Table 1 and Figure 6).

##### Continuous endurance exercise protocols

Over the thirteen studies that involved the application of ice or cold beverages before a continuous endurance exercise protocol, five observed significant improvements in physical performance. Completion time (0.6-7.2%) and power output (5.4-7.8%) were significantly enhanced in a 40-km (96) and 800-kJ (124) cycling time trials, as well as in a 10-km running time trial (104). Total distance was also improved in a 30-min cycling time trial protocol (2.9%) (94), and time to exhaustion was significant higher in a running to exhaustion at first ventilatory threshold in a treadmill (12.8%) (114). However, time to exhaustion was not significantly improved in a 80% VO_2_max speed cycling protocol (131), neither mean power output or total work done in a 60-min fixed 15-RPE (121) and 60-min fixed 55%VO_2_peak cycling protocols (115). Finally, completion time was not significantly lower while pre-cooling with ice or cold beverages in a 30-km (123), 800-kJ (98) and 30-min fixed 75% of peak power output (125) cycling time trials, not either in a 5-km running time trial protocol (129).

In the nine studies involving per-cooling with ice or cold beverages on continuous endurance exercise protocols, five returned significant enhancements on physical performance. Completion time (10.5%) was significantly improved in a cycling time trial protocol with a resistance of 4 kJ kg^–1^ (133), as well as running time (2.5%) in a triathlon (Olympic distance) protocol (126), and work done (4.4%) in a 15-min maximal intensity cycling protocol (95). Time to exhaustion (7-12.7%) was significantly superior in a 65% (112) and 70% (116) maximal aerobic power output cycling protocols. Thought, time to exhaustion and endurance capacity were not significantly improved in cycling at 50% (139) and 95% (118) VO_2_peak intensity, neither in a running to exhaustion at first ventilatory threshold intensity (113). On the other hand, completion time was not significantly lower in a 40-km cycling time trial (109).

In the set of four studies where ice or cold beverages were administered both before and during continuous endurance exercise, all showed significant improvements in physical performance. Significantly higher time to exhaustion (23-118%) was observed in a 80% of maximum power intensity and in a 65%VO_2_peak cycling protocols (107, 120), and completion time (6.2-6.8%) was also significantly improved in 20-km cycling (108) and in a 1.5-km running (130) time trials (Supplementary Table 1 and Figure 6).

##### Influence of environmental conditions

Generally, thirteen out of twenty-nine studies that were performed under a WBGT ≥28°C and involved the internal administration of ice or cold beverages revealed significant improvements in physical performance (94–96, 107, 108, 112, 114, 116, 120, 124, 126, 133). When the analysis is differentiated by type of exercise, it is clear that all the thirteen studies involved continuous endurance exercise protocols. In the range of the studies that did not show significant improvements in physical performance while a thermal cooling technique was applied in more adverse environmental conditions, six involved intermittent exercise protocols (93, 99, 119, 122, 127, 132) and ten continuous endurance ones (98, 105, 109, 113, 115, 121, 123, 125, 129, 131).

**FIGURE 7:**
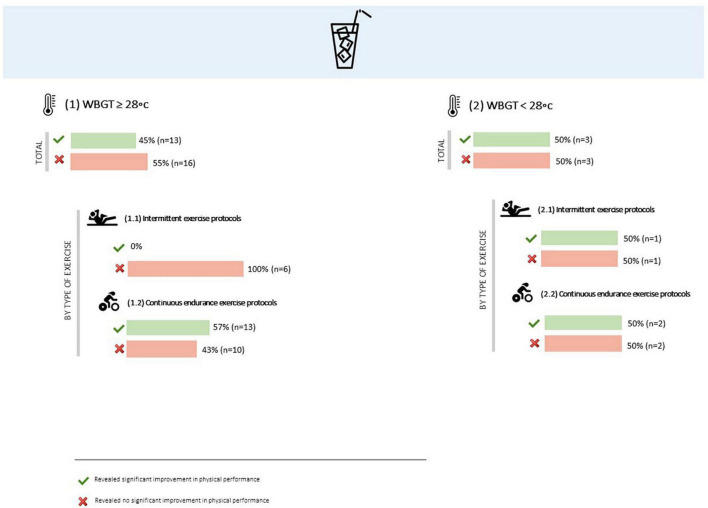
Overall results of ice or cold beverages administration effect on physical performance, by environmental conditions and type of exercise.

Moreover, three out of six studies that involved the administration of ice or cold beverages under a WBGT <28°C showed significant improvements in physical performance. Two of them included continuous endurance exercise protocols (104, 130) and one included intermittent exercise (134). In the set of the studies that did not find significant improvements in physical performance, one involved intermittent exercise protocols (97) and two continuous endurance ones (118, 139) (Figure 7).

#### Ice *vs.* cold beverage

##### Continuous endurance exercise protocols

One study (130) showed that ingesting ice slurry both before and during exercise significantly decreased completion time in a 1.5-km running time trial (3.3%) comparing to drinking a cold beverage at the same timings (Supplementary Table 1).

#### Menthol solution *vs.* ice or cold beverage

##### Continuous endurance exercise protocols

In the three studies where the ergogenic effect of oral menthol administration was compared to ice or cold beverages, two showed a significant improvement in physical performance with the non-thermal technique, compared to the thermal one. Relative power output was significant higher (6%) while pre-cooling with menthol thermoneutral solution in a 3-min aerobic test compared to pre-cooling with cold water (105). Simultaneously, other study observed that per-cooling with a menthol solution had significant benefits on completion time in a 5-km running time trial (3.8%), compared to pre-cooling with an ice-slurry (129).

On the other hand, per-cooling with a menthol solution at 85% of baseline time to exhaustion showed non-significant differences in physical performance in a 70% maximal aerobic power output cycling protocol, compared to per-cooling at the same time with an ice slurry (116) (Supplementary Table 1).

#### Mixed-method *vs.* non-cooling strategy

##### Continuous endurance exercise protocols

Mouth rinsing with a menthol solution during exercise and ingesting crushed ice before exercise showed a significant increase in time to fatigue (39.1%) in a running protocol at an intensity of 100% of VO_2_peak, comparing to a non-cooling control (128). At the same time, consuming an ice slushy menthol flavored beverage before and during a 20-km cycling time trial revealed a significant decrease in completion time (11%), comparing to a non-cooling strategy (108) (Supplementary Table 1).

## Discussion

The current systematic review aimed to determine the effects of internal thermal and non-thermal cooling strategies on physical performance in different exercise conditions. The main findings were that mouth rinsing with a menthol solution during continuous endurance exercise in the heat seems to significantly improve physical performance in most of the studies. The impact of thermal methods on physical performance is not consistent, with only less than half of the studies reporting improvements. Ice/cold beverages are more likely to enhance physical performance in continuous endurance exercises, especially when consumed throughout exercises.

### Menthol

#### Ergogenic action and physiological mechanisms

Oral menthol mouth rinsing during exercise in the heat improved continuous exercise, either exhaustive (111, 116, 128), self-modulated to exhaustion over a fixed distance or time (time-trial) (100, 108, 129), or to a fixed RPE (101, 110), which is in line with the recent findings of Barwood and colleagues (138).

Menthol’s ergogenic effects seem to be related with an improvement in thermal sensation (101, 108, 110, 116), rate of perceived exertion, ventilatory capacity (111, 129), brain capacity (100), increased glycolytic energy provision or increased bicarbonate buffering (105). Menthol works as a non-thermal cooling stimulus to thermoreceptors, eliciting sensations of coolness when applied to the skin and mucosal surfaces without reductions in temperature, enhancing perceptual cooling effects (140, 141). Concretely, the application of menthol cause a feeling of coolness due to a stimulatory action on “cold” receptors (primarily TRPM8) by inhibiting calcium currents of neuronal membranes (142). The activation of these sensory pathways conducts this information to the brain, lowering the associated thermal strain (143), and may also provide a decrease in subjective airway resistance, a sensation of cool airflow upon inhalation, an increased arousal, and a down-regulation of thirst (142).

Despite menthol did not seem to increase tidal volume, subjects voluntarily increased ventilation following the rinse, perhaps due to the sensation of cooler air (111). A lowered thermal sensation may allow athletes to undergo greater heat stress, through increased blood prolactin concentration (129). Prolactin has been described as a “stress hormone” and a marker of dopaminergic activity in the brainstem (144). It is released in response to increased heat load (145), more specifically regarding the temperature of the facial surface during exercise (146). A higher prolactin concentration suggests that following the menthol solution rinse the body can tolerate greater stress associated with a higher exercise intensity. For this reason, it is not clear if a decrease in thermal sensation can lead to a greater fatigue or even to a higher risk of hyperthermia, especially in the latter stages of prolonged exercise in elite athletes (110, 129).

Two of the three studies included in the present systematic review in which menthol did not improve performance applied intermittent exercise protocols (103, 106). It is important to mention that the conditions were less demanding in these studies, either by being held in a normothermic environment (22 ± 1°C) (106) or due to a shorter exercise protocol (20 min) and a greater recovery period between sets (103). Also, even if a cycling intermittent-sprint protocol is a reliable and valid tool to determine the physiological responses to intermittent sprinting (147), the specific task being closed in nature could create an experimental artifact, whereby participants are not able to sprint freely in frequency or duration. This raises the need to assess the effect of oral menthol under an intermittent exercise protocol that more closely mimics real conditions. In another study, in which menthol did not significantly enhance the time to exhaustion in a continuous endurance exercise protocol (117), the trial was not performed under heat stress conditions (20.4 ± 0.5°C, 29.5 ± 4.6% RH, WBGT = 18°C). Additionally, this was the only protocol to incorporate menthol into a carbohydrate sports drink, while the control beverage being the same beverage without menthol. Although speculative, the lack of significant benefits on physical performance in this study could be due to the fact that the sensing of carbohydrates and menthol in the mouth affects similar nervous signaling pathways and, hence, the erogenicity of both substances is not additive. In fact, carbohydrate mouth rinsing is known to be ergogenic on its own, without supplying additional energy to the body (148). Therefore, more significant results are found with the administration of menthol, in comparison with no-beverage or beverages that do not exert an ergogenic role.

#### Timing and administration mode

Overall, the collective evidence presented in the current systematic review suggests that menthol does not need to be swallowed to elicit a positive effect on performance, being sufficient to orally rinse and expectorate (100, 101, 105, 110, 111, 116, 128, 129). Menthol activates thermoreceptors located in the oral cavity, one of the most densely innervated parts of the body with several peripheral receptors (149), that my trigger an ergogenic effect (108).

Almost all studies conducted with oral menthol have opted for a per-cooling strategy, which seems more suited to its mode of action. As the proposed mechanism of action of internally applied menthol targets thermal sensation, improved thermal comfort, and rate of perceived exertion (15, 150), the ergogenic effect is more likely to occur with the higher thermal stress obtained in more advanced stages of exercise. Accordingly, menthol seems to benefit performance when given in several successive internal administrations throughout exercise (100, 101, 108, 110, 111, 128, 129) or even as a single dose in the latter stages of exercise (116).

Most studies (100, 105, 110, 116, 128, 129) have demonstrated a beneficial effect on physical performance with a mouth rinsing for 5 s, without no greater effect observed with longer rinse durations (up to 10 s) (116, 117).

#### Sex differences

Five studies have evaluated the role of oral menthol on physical performance in female participants (100, 101, 103, 105, 106), and two of them reported significant improvement in exercise (100, 105). However, three of the five studies did not present the results separated by sex, which makes impossible to differentiate the effect of oral menthol mouth rinse on physical performance in men and women, no matter it is significant (105) or not (103, 106). In the other two studies, one found significant results with cooling only for men (101) and the other observed a significant improve in completion time and power output in the female subjects (100). Therefore, is not clear if the lack of efficacy on women depends on sex-related physiological differences, or if is due to methodological differences between studies.

Interestingly, oral application of L-menthol reduced the perceptual measures of thermal sensation in men, while in females it was only effective in the early stages of exercise in the heat. Women exhibited a smaller reduction in thermal sensation following L-menthol mouth rinsing, suggesting a sex-specific response to L-menthol efficacy during exercise (101). Mouth rinsing with 25 mL (0.01%) every 10 min of exercise improved non-significantly the time (6%) and power output (2.2%) in women (101). Therefore, these facts highlight the importance of further research to understand sex differences in behavioral thermoregulation and performance response to oral menthol during exercise in the heat.

#### Influence of environmental conditions

Only one study involving oral menthol administration was carried out under WBGT <28°C and this included continuous endurance exercise protocol (117), revealing no significant improvements in physical performance. In fact, as previously mentioned, in this study menthol was incorporated into a carbohydrate sports drink, possibly not allowing to isolate the ergogenic effect of menthol from that of carbohydrates. Also, the same study was carried out under a WBGT = 18°C, which represents a low risk of overheating environment (137). Therefore, the fact that the only study that included menthol and was carried out under a WBGT <28°C had methodological characteristics so different from the others, makes it difficult to conclude about the role of the environmental conditions. More studies developed under a WBGT <28°C with non-thermal internal cooling techniques are needed to better conclude if these techniques are also efficient in physical performance, under these conditions.

It is important to consider that, in the case of menthol, although more studies have been completed under a WBGT ≥ 28°C, the type of exercise seems to be more important than the adversity of the environmental conditions, since the only study that did not revealed significant improvements in physical performance in these conditions involved an intermittent exercise protocol (103).

As already stated, performance responses to internal cooling seem to be better in continuous endurance exercise than in intermittent exercise. In fact, in continuous exercise, the heat stored is higher and T_core_ and thermal sensation rise faster, compared to the same amount of exercise performed in a variable intensity mode that includes short high intensity bouts followed by rest periods (151–153). Also, sweat loss and dehydration level are lower in intermittent compared to continuous type sports (154). Therefore, regardless of the external environmental conditions, it is expected that in activities where the heat storage and dehydration turns out to be superior, the response to internal cooling will be more effective concerning physical performance, as improvements in T_core_ or thermal sensation are more urgent. Since menthol’s main mechanism of action is an improvement in the thermal sensation in more advanced phases of the exercise, if this is higher it is more likely that the ergogenic effects of this compound, previously explained, are felt.

### Ice or cold beverage

#### Ergogenic action and physiological mechanisms

The application of thermal methods before and/or during exercise improved performance in 15 of 35 studies that evaluated exhaustive (time to exhaustion at a fixed intensity) (107, 112, 114, 116, 120, 131), self-modulated to exhaustion over a fixed distance or time (time trial) (94, 96, 104, 108, 124, 126) or to a fixed point (power output) (95, 133), and intermittent protocols (93, 134). An improvement in performance was more prevalent in studies evaluating continuous endurance exercise (54%), than in studies that applied intermittent exercise protocols (11%).

Overall, the improvement in physical performance with the internal administration of ice or cold beverages may have resulted from an improve in behavioral thermoregulation ((thermal sensation (104, 130), thermal comfort (120)), thermal autonomic responses ((T_core_ (94–96, 107, 112, 134)), or from a combination of all these (114, 124, 126, 133). After the ingested ice slurry reaches the stomach/gastrointestinal region it will absorb a considerable amount of internal heat, lowering the temperature locally (96). It is also likely that the ingestion of ice may also lead to a decrease in brain temperature (155), that may persist throughout exercise, increasing the likelihood of an improvement regarding thermal sensation in the latter stages. This may extend the time required to achieve a critically high brain temperature, allowing subjects to exercise for longer periods of time or greater intensities (53, 114, 156, 157), by increasing and/or maintaining central drive and motivation (158). In addition, internal cooling via ice slurry ingestion may improve exercise performance in the heat by stimulating internal thermoreceptors. In humans, thermoreceptors have been identified in the stomach and small intestine (159) It has been shown that the glossopharyngeal nerve conducts impulses for temperature sensation from the posterior third of the tongue and upper pharynx to the brain (160). Thus, ice slurry ingestion may directly affect T_core_ afferents and leads to a beneficial effect on the inhibitory feedback, ultimately influencing exercise performance. A decrease in T_core_ may affect exercise performance by increasing the margin between the initial core temperature and temperatures at which athletic performance is affected. A lower core body temperature at a given point of exercise had a similar effect to that which occurs with acclimation and enabled athletes to exercise at higher intensities during self-paced exercise (or for a longer duration during constant pace exercise) (161). In fact, a decrease in T_core_ is likely to promote a reduction in sweat rate necessary for heat dissipation, delaying progressive fluid losses and dehydration (162). Progressive dehydration precipitates a cascade of events including a decrease in plasma volume and an increase in plasma osmolality (5), a decrease in sweat rate and evaporative heat loss (163) and a decrease in cardiac filling (164). The blood flow redistribution and other thermoregulatory demands of exercising in hot and/or humid environments represents a significant stress to the cardiovascular system, limiting performance, as maintaining a similar relative intensity requires the reduction of absolute intensity (i.e., work load) (165).

Putative reasons for the lack of performance improvements with internal cooling strategies in some studies may be related with a lower core-to-skin temperature gradient (121, 123), no reduction on core body temperature (T_core)_, (i.e., lower heat storage capacity) (119), self-paced intermittent protocols (99, 122, 127, 132), short duration protocols (97, 131), moderate environmental conditions (97, 139), no changes in thermal sensation and rate of perceived exertion (107, 115, 139), or gastrointestinal discomfort due to an excessive amount of ice slurry (125). Actually, a lower core-to-skin temperature gradient found in two studies (121, 123) did not promote a sufficient convective heat flux from the center to the periphery, hindering thermoregulation mechanisms to cope with the heat and thus not benefiting performance. This can happen when the environmental temperature is much higher than skin temperature, and also when T_core_ decreases very fast at the beginning of the exercise (93). On the other hand, cooling strategies may provide the most gains in physical performance in stressful environmental conditions (higher temperatures and humidity levels and/or longer duration and greater intensity exercises) (95), that were not observed in some of the included studies (97, 99, 122, 127, 131, 132, 139). As increases in core temperature are proportional to exercise intensity (166), it is easier to avoid an increase in heat strain in exercises protocols whose intensity is not great, or at least is interspersed with moments of lower intensity recovery periods (intermittent efforts), minimizing the benefits of administering an ice or cold beverage. At the same time, in other studies (107, 115, 139), authors report that, despite the improvement observed in the thermoregulatory parameters (such as T_core_), physical performance was not increased due to a lack of improvement in performance perceptual parameters (thermal sensation and rate of perceived exertion). This could indicate that behavioral thermoregulation and thermal autonomic responses to exercising in the heat are equally important, and that the success of cooling strategies may depend on its effectiveness in improving both variables. Finally, it is known that the maximum rate of intestinal absorption is 0.5 L/hour when cycling at 85% VO_2max_ and the intake of large volumes of fluids may not be advantageous (167). This could be the reason why administrating 1 L of ice-slurry at once found no improvements on physical performance, despite a reduction on T_core._ in one study (125).

#### Timing and administration mode

Ingesting ice or cold beverages seemed to be more effective in improving physical performance than just rinsing, which may be explained by the higher density of thermal receptors in the gastrointestinal tract (12).

The amount of ice or cold beverages administered before and/or during exercise was quite different between trials. Also, some authors opted for giving an absolute amount, while others preferred to adjust the volume to athletes’ body weight (g⋅kg^–1^) due to an improved gastrointestinal tolerance (94, 130, 133). However, performance benefits were observed with a wide range of volumes, which suggest that the amount ingested is not a key factor regarding the ergogenic potential of these beverages.

Ice or cold beverages showed better results when administered during or before and during exercise (93, 95, 107, 108, 112, 116, 120, 126, 133). Per-cooling protocols involved the ingestion of ice slurry/crushed ice/ice slushy/cold beverages several times throughout exercise, while in the pre-cooling protocols these beverages were ingested once, typically between 10 to 35 min before the beginning of exercise (94, 96, 98, 99, 104, 114, 115, 121–125, 127, 129, 131, 134). Some recent studies suggested that the advantages gained from cooling during exercise may outweigh those of pre-cooling, due to a high thermal strain in the latter stages of the exercise (168). Although, T_core_ seems to decrease more with pre-cooling (physical thermoregulation), per-cooling seems to be more efficient in reducing thermal sensation and thermal comfort (behavioral thermoregulation) in more advanced phases of the exercise (12). So, the effectiveness of pre-cooling with ice slurry may be limited and its beneficial effects may be attenuated after 20–30 min (169).

Regarding beverage’s temperature, it was found that ingesting 190 mL of ice slurry menthol flavored beverage (0.025%, 0.17°C) in the warm-up and 5 times during running, significantly decreased completion time (3.3%) when comparing with the same amount of cold menthol flavored beverage (0.025%, 3.1°C) (130). This agrees with another study (50) reporting that the ingestion of 1.25 g⋅kg^–1^ of ice (0.5°C) every 5 min, six times before exercise, significantly improved time to exhaustion (19.8%), when comparing with the same amount of cold water (4°C). This difference may have occurred due to different effects on T_core_, since crushed ice ingestion leads to a greater reduction in body temperature due to the additional energy that is required to change solid ice to liquid water, allowing for a significantly larger amount of heat absorption and thus more work to be completed (53).

#### Sex differences

The only study (of five) with participants from both sexes that reported a significant improvement in physical performance with internal thermal cooling techniques in a hot and humid outdoor environment, enhancing the ecological validity of the findings, did not independently report the effect of ice slurry ingestion 30 min before exercise (8.0 g⋅kg^–1^ at −1.4°C) for men and women (104). In other studies, the consumption of either cold water during exercise (190 mL at 1.3°C) (102) and of crushed ice 30 minutes before exercise (6.8 g⋅kg^–1^ at −0.5°C) (99), did not change physical performance. However, it should be mentioned that one of that studies (102) comprised swimming and exercise in water, which increases the heat dissipation capacity due to greater forced convective and conductive transfer heat transfer from the skin (170), facilitating the maintenance of T_core_ at a lower level and thus reducing the likelihood of cooling benefits. And int the other study (99), the intermittent exercise protocol used was less affected by cooling strategies. Similarly, mouth rinse of cold water 1 min before the start of exercise (25 mL at 4°C) did not show effect on relative power output in both male and female subjects (105). Though, it is important to refer that applying a thermal cooling technique only 1 min before a 3-min exercise may not be enough time to notice the benefits of these methods, neither for men nor for women. Finally, another trial (98) showed a non-significant decrease in completion time (2.2%) in a 800-kJ cycling time trial in women after the ingestion of crushed ice (7.0 g⋅kg^–1^) 30 min before. It is expected that an athlete with a higher percentage of fat mass requires less energy to change their mean body temperature when comparing with an athlete with a lower percentage of fat mass (171). Therefore, as the rate of heat storage may be greater in females than in males, this might hinder the ergogenic effect due to an increased heat storage capacity, which could explain why ice ingestion may exert greater benefits in men.

#### Influence of environmental conditions

Unlike menthol, slightly better results were found on physical performance when using ice or cold beverages under a WBGT <28°C, compared to the studies carried out in a WBGT ≥ 28°C. However, it is important to notice that all the studies completed with a WBGT ≥ 28°C that showed significant improvements in physical performance (*n* = 13) involved continuous endurance exercise protocols (94–96, 107, 108, 112, 114, 116, 120, 124, 126, 133, 137). Concerning the other sixteen studies carried out under a WBGT ≥28°C, and that did not reveal significant enhancements, six involved intermittent exercise protocols (67, 93, 99, 113, 119, 122, 127) and ten continuous endurance ones (98, 105, 109, 114, 115, 121, 123, 125, 129, 131). Of these ten studies that involved endurance exercise, eight had a pre-cooling mode (98, 105, 115, 121, 123, 125, 129, 131), which, as seen in point 4.2.2, will eventually not be the most interesting cooling timing for thermal methods.

Like menthol, more important than the environmental conditions seems to be the type of exercise protocol. As mentioned before, in continuous exercise, the heat stored is higher and T_core_ and thermal sensation rise faster. Thus, this type of exercise, regardless of environmental conditions, will theoretically benefit better from thermal cooling methods than intermittent exercises, especially those of shorter duration.

This may indicate that the benefits of cooling on physical performance are not only verified for high or extreme environmental conditions, and that, even in less demanding conditions, for higher and continuous exercise intensities, it could still be beneficial the application of internal cooling techniques. Nevertheless, further studies involving simultaneously internal cooling thermal techniques, continuous endurance exercise and per or pre + per-cooling modes, both in more and less adverse environmental conditions, will be needed for a better understanding.

### Mixed thermal and non-thermal techniques

Combining thermal with non-thermal strategies seems to display better results than an isolated approach. As an example, superior results on completion time were reported when thermal and non-thermal techniques were combined (ice slurry menthol flavored – 0.01%), instead of used in isolation (108). Similarly, the combination of pre-cooling with ice slurry ingestion and per-cooling with menthol solution mouth rinse improved time to fatigue compared to per-cooling with menthol alone (128). A possible reason to justify these results is the reduction of the mood disturbance, in particular ratings of tension, depression, and confusion, when thermal and non-thermal methods are simultaneously applied (128). However, a synergistic effect of thermal and non-thermal interventions should be considered in future research, for a better understanding.

### Menthol solution *vs.* ice or cold beverage

Although two of the three studies that compared the effect of menthol with ice or cold beverages found greater benefits in physical performance with the non-thermal method, it is important to note that in one of them (105) the pre-cooling (either with menthol or with a cold beverage) was carried out just 1 min before the trial and that the trial itself only lasted 3 min, not sufficient to see a decrease in T_core_. In the other study (129), while menthol was applied as per-cooling, ice slurry was ingested 30 min before exercise. Thus, in this case, we may be observing the benefits of applying a cooling method during the exercise (which, as previously mentioned, seems to be more advantageous than pre-cooling), rather than comparing the effectiveness of a thermal method with a non-thermal one.

## Limitations and strengths

This systematic review has some limitations. Firstly, most studies included a relatively small sample size. Secondly, men and women seem to have different thermoregulatory responses to exercise in the heat, which may have influenced the effectiveness of cooling methods on physical performance, especially regarding mixed-sample studies. Another possible limitation is related with the fact that trials were performed in an ambient environment with large temperature and relative humidity amplitudes (i.e., 22-38°C and 20-80%). Moreover, exercise protocols noticeably varied between studies, namely regarding duration and intensity, possible generating conflicting results. Furthermore, authors opted to not collect adverse effects in the selected studies. In fact, in all the included studies, a familiarization session was performed to test participants tolerance to L-menthol or to ice/cold beverages, and any participant has been excluded from any study based on intolerances or adverse effects. Additionally, the included studies did not evaluate immediate/long term complications resulting from the intervention, so for those reasons adverse effects were not reported in the data synthesis. Other limitation is that with 43 studies and 472 subjects included, a meta-analysis could potentially have been carried out. However, due to the high heterogeneity of the methodologies used, not only regarding the systematic differences of exercise protocols, but also considering the variances in timing and frequency of cooling, in the beverages doses, in the population involved, and in the environmental conditions, the authors opted not to perform a quantitative analysis. Additionally, the risk of bias in the selective reporting and blinding categories was generally unclear. In fact, this may be difficult to assure when administering menthol or ice/cold beverages, due to its distinctive sensory effect. Whilst it is challenging to conduct double-blind experiments in this type of studies, improvements in research design, mainly with menthol, should be attained. Finally, publication bias was not accessed in this systematic review.

Regarding the strengths of this article, we first highlight the fact that, to the best of our knowledge, this is the first systematic review that exclusively focuses on internal cooling methods. The literature is mainly focused on external techniques. Secondly, only studies with a randomized crossover design were included, increasing the internal validity of the results (172). Furthermore, this review involved studies with several types of exercise protocols, which allows for a wider broad understanding of the internal cooling effect on physical performance in different sports disciplines, considering their physiological particularities. It is also important to notice that this analysis, unlike most others that focus exclusively on pre or per-cooling modes, was able to conclude about the cooling timing role on physical performance.

## Future lines of research

Sport competitions are taking place more often in hot environments, so the implementation of cooling techniques before and during competition will become even more important for athletes to cope with the heat. More solid evidence is needed about the implication of cooling strategies on short and intense and even intermittent efforts, especially in outdoor settings and that approach the real conditions and rules of different sports, including team sports. Moreover, studying both male and female individuals is of upmost importance, due to sex-related differences in thermal sensation and heat storage capacity. Since the majority of the collected evidence in this topic refers to physically active individuals or recreational athletes, protocols that involve elite athletes are also required. Likewise, it is also important to understand the effect of different environmental conditions on the response to internal cooling, so that the recommendations can take this factor into account.

## Practical applications

In addition to acclimatization or in situations where this is not feasible, athletes competing in outdoor higher temperatures and humidity contexts should adopt prophylactic strategies to avoid adverse effects resulting from a temperature rise of body temperature on physical performance. Such strategies may be practical and cost-effective, namely the ingestion of an ice slurry 30 min before exercise and the ingestion or mouth rinsing of an ice slurry menthol flavored beverage (0.01%) during exercise. In endurance exercise competitions, per-cooling strategies can be divided into multiple moments during the race, including hydration breaks, half-time or drinking stations.

## Conclusion

Rinsing a menthol solution (0.01%) improves physical performance during continuous endurance exercise. Conversely, the ingestion of ice or cold beverages does not seem to increase performance. However, slightly greater results were found for ice beverages, in per-cooling or pre and per-cooling continuous endurance trials. Co-administration of menthol with or within ice beverages seems to exert a synergistic effect by improving physical performance. Although not entirely clear, even in environmental conditions that are not extreme, internal cooling strategies may exert an ergogenic effect. Further studies exploring both intermittent and outdoor exercise protocols, involving elite male and female athletes and performed under not extreme environmental conditions are warranted.

## Data availability statement

The raw data supporting the conclusions of this article will be made available by the authors, without undue reservation.

## Author contributions

MR and VT were responsible for conception and design. MR and PB conducted search procedure, data analysis, and methodological quality analysis of the included studies. All authors made substantial contributions to conception, design, and interpretation of the data, drafting of the manuscript, and in giving final approval of the final version, read, and agreed to the published version of the manuscript.
