# Supplementary material for: Performance effects of internal pre- and per-cooling across different exercise and environmental conditions: A systematic review
Source: Front Nutr. 2022 Oct 14;9:959516. doi: 10.3389/fnut.2022.959516 (PMC9632747; doi:10.3389/fnut.2022.959516)
Supplement: Supplementary file 1 [file Data_Sheet_1.PDF]

frontiers

Appendix A. Search Strategy for Pubmed, Scopus, Web of Science and SportDiscus

Pubmed (303 citations)

Search performed: April 4, 2022

#1	“athletic performance” [Mesh] (60,470)
#2	“sports performance” [tiab] (1,396)
#3	“physical activity” [tiab] (133,811)
#4	“exercise” [tiab] (301,155)
#5	“athletes” [tiab] (54,563)
#6	“sports” [tiab] (62,250)
#7	#1OR#2OR#3OR#4OR#5OR#6 (500,255)
#8	“cooling” [tiab] (44,622)
#9	“pre-cooling” [tiab] (212)
#10	“per-cooling” [tiab] (17)
#11	“mid-cooling” [tiab] (10)
#12	“body temperature” [tiab] (34,907)
#13	“heat mitigation” [tiab] (69)
#14	#8OR#9OR#10OR#11OR#12OR#13 (77,818)
#15	“ice slurry” [tiab] (124)
#16	“ice slushy” [tiab] (13)
#17	“ice slush” [tiab] (130)
#18	“cold water” [tiab] (5,231)
#19	“cold fluid” [tiab] (126)
#20	“beverage” [tiab] (15,154)
#21	“drinking” [tiab] (124,696)
#22	“menthol” [tiab] (3,530)
#23	“mint” [tiab] (1,961)
#24	“peppermint” [tiab] (1,451)
#25	#15OR#16OR#17OR#18OR#19OR#20OR#21OR#22OR#23OR#24 (149,552)
#26	#7 AND #14 AND #25 (303)

Scopus (813 citations)

Search performed: April 4, 2022

1	[TITLE-ABS-KEY] “Exercise” OR “Athletes” OR “Sports” OR "Physical Activity" OR “Sports Performance” OR “Athletic performance”	(1,111,087)
2	[TITLE-ABS-KEY] "Body temperature" OR "Heat mitigation" OR “Cooling” OR “Pre-cooling” OR “Per-cooling” OR “Mid-cooling”	(605,556)
3	[TITLE-ABS-KEY] “Drinking” OR “Beverage” OR "Cold fluid" OR "Cold water" OR "Ice slurry" OR “Ice-slush*” OR “Menthol” OR “Mint” OR “Peppermint”	(398,743)
4	1 AND 2 AND 3	(813)

Web of Science (636 citations)

Search performed: April 4, 2022

#1	“Exercise” OR “Athletes” OR “Sports” OR "Physical Activity" OR “Sports Performance” OR “Athletic performance”<br><br>https://www.webofscience.com/wos/woscc/summary/58abf12b-b28a-41bd-b0a7-8d8b8e665161-33bc1ac0/relevance/1	(1,542,309)
#2	"Body temperature" OR "Heat mitigation" OR “Cooling” OR “Pre-cooling” OR “Per-cooling” OR “Mid-cooling”<br><br>https://www.webofscience.com/wos/woscc/summary/b5b7e6c7-c8dd-44e5-bf27-bbb280f17ecd-33bc2767/relevance/1	(361,754)
#3	“Drinking” OR “Beverage” OR "Cold fluid" OR "Cold water" OR "Ice slurry" OR “Ice-slush*” OR “Menthol” OR “Mint” OR “Peppermint”<br><br>https://www.webofscience.com/wos/woscc/summary/99f0a585-c89e-4bd7-bdba-9e8647fb8c1f-33bbf83a/relevance/1	(258,713)
#4	#1 AND #2 AND #3<br><br>https://www.webofscience.com/wos/woscc/summary/d012b72e-3363-44fa-a3b8-2347bbf77172-33bc5641/relevance/1	(636)

SportDISCUS (962 citations)

Search performed: April 4, 2022

1	“Exercise” OR “Athletes” OR “Sports” OR "Physical Activity" OR “Sports Performance” OR “Athletic performance”	(3,140,283)
2	"Body temperature" OR "Heat mitigation" OR “Cooling” OR “Pre-cooling” OR “Per-cooling” OR “Mid-cooling”	(299,027)
3	“Drinking” OR “Beverage” OR "Cold fluid" OR "Cold water" OR "Ice slurry" OR “Ice-slush*” OR “Menthol” OR “Mint” OR “Peppermint”	(862,716)
4	1 AND 2 AND 3	(962)
