# Supplementary material for: Performance effects of internal pre- and per-cooling across different exercise and environmental conditions: A systematic review
Source: Front Nutr. 2022 Oct 14;9:959516. doi: 10.3389/fnut.2022.959516 (PMC9632747; doi:10.3389/fnut.2022.959516)
Supplement: Supplementary file 2 [file Data_Sheet_2.pdf]

Table 1. Summary of the included studies with a continuous endurance exercise protocol.

Study	Ambient conditions	Participant characteristics	Design	Exercise protocol	Cooling timing	Cooling technique	Performance outcomes
**Menthol**							
Flood et al., 2017 (110)	35 ± 0.8°C<br>47.8 ± 2.3% RH	Non-acclimated male (n=8).<br>Age 26 ± 5 y.<br>VO_2max 55.4 ± 6.0 mL·kg^{-1}·min^{-1}	Crossover, randomised, single-blind	5 s isokinetic sprint + fixed 16-RPE cycling	Per-cooling	25 mL L-menthol solution mouth rinse (0.01%, 5 s, 19.8±0.4°C) 1.5 min before fixed RPE and every 10 min	**↑7% T (p=0.049)**<br>**↑3.6% PO (p=0.044)**
Gavel et al., 2021 (100)	30 ± 0.6°C<br>70.0 ± 1.0% RH<br>12.2 ± 1.2 km h^{-1} WS	Non-acclimated trained female (n=9).<br>Age 26.7 ± 1.4 y.<br>VO_2max 50.8 ± 6.0 mL·kg^{-1}·min^{-1}	Crossover, counterbalance, randomised	30-km cycling independent TT	Per-cooling	25 mL L-menthol solution mouth rinse (5s,>22°C) at 5-km intervals	**↓2.3% TT (p=0.034)**<br>**↑6.0% PO (p=0.031**
Mundel and Jones 2010 (111)	34 ± 1°C<br>27 ± 4 % RH	Healthy non-acclimated males (n=9). Age 25 ± 7 y.<br>VO_2max 54 ± 5 mL·kg^{-1}·min^{-1}	Crossover, randomised	TTE at 65%W_{max}	Per-cooling	25 mL L-menthol solution mouth rinse (0.01%, 10 s, 19±2°C) every 10 min	**↑8.6% TTE (p=0.043)**
Parton et al., 2020 (101)	34.9 ± 0.5°C<br>40.6 ± 2.2% RH	Non-acclimated male (n=11).<br>Age 20 ± 1 y.<br>VO_2peak 53.9 ± 6.9 ml kg^{-1} min^{-1}.<br>Non-acclimated female (n=11).<br>Age 22 ± 2 y.<br>VO_2peak 43.5 ± 2.9 mL·kg^{-1}·min^{-1}	Crossover, randomised, double-blind	Cycling at FI 16-RPE	Per-cooling	25 mL menthol solution mouth rinse (0.01%, 10 s, 31.8°C) 30 s before exercise and every 10 min	Male<br>↑4% T (p>0.05)<br>**↑6.5% PO (p=0.039)**<br>Female<br>↑6% T (p>0.05)<br>↑2.2% PO (p=0.475)

Table 1. (continued)

Study	Ambient conditions	Participant characteristics	Design	Exercise protocol	Cooling timing	Cooling technique	Performance outcomes
Podlogar, et al., 2021 (117)	20.4 ± 0.5°C<br>29.5 ± 4.6% RH	Healthy, endurance-trained male (n=12).<br>Age 29 ± 5 y.<br>VO_2peak 57.3 ± 6.4 ml kg $^{-1}$ min^{-1}	Crossover, counterbalance, randomised	Cycling for 60 min at 90% VT1 + TTE at 105% RCP	Per-cooling	85 mL menthol-containing sports drink mouth-rinse (0.01%, 5-10 s, 20°C) every 10 min	↑4.4% TTE (p>0.05)
**Ice/Cold Beverages**							
Barwood et al., 2018 (121)	WBT<br>21.7 ± 0.9°C<br>33.9 ± 1.4% RH<br>2.8 ± 0.3 m s $^{-1}$ WS<br>DBT<br>34.4 ± 0.7°C	Trained, non-acclimated males (n=12).<br>Age 25 ± 5 y	Crossover, randomised	Cycling: 60 min of FI 55% P_{Max}+ 80% P_{Max} TTE	Pre-cooling<br>Per-cooling	3.2 mL kg^{-1} cold beverage ingestion (5°C) 10 min before exercise<br>+<br>3.2 mL kg^{-1} cold beverage ingestion (5°C) every 15 min within 45 min	**↑118% TTE (p=0.021)**
Burdon et al., 2010 (95)	28°C<br>70% RH<br>3.6 km h^{-1} WS	Non-heat acclimated male cyclists (n=7).<br>Age 32.8 ± 6.1 y.<br>VO_2peak 59.4 ± 6.6 mL·kg $^{-1}$·min^{-1}	Crossover, randomised	90 min 65% VO_2 peak + 15 min maximal intensity cycling	Per-cooling	2.3 mL kg^{-1} cold beverage ingestion (4°C) every 10 min starting at 10 min of exercise	**↑4.4% W (p=0.004)**
Burdon et al., 2013 (134)	32°C<br>40% RH<br>3.6 km h^{-1} WS	Healthy naturally acclimatized male endurance cyclists (n=10).<br>Age 30.1 ± 7.0 y.<br>VO_2max 61.8 ± 5.6 mL·kg $^{-1}$·min^{-1}	Crossover, counterbalance, randomised	90 min of steady-state cycling at 62% VO_2peak + TT with a resistance of 4 kJ kg^{-1} until exhaustion	Per-cooling	260 g ice slushy ingestion (-1°C) every 15 min of SS exercise	**↓10.5% TT (p<0.05)<br>↑10.7%PO (p<0.05)**

Table 1. (continued)

Study	Ambient conditions	Participant characteristics	Design	Exercise protocol	Cooling timing	Cooling technique	Performance outcomes
Byrne et al., 2011 (94)	DBT<br>32°C<br>60% RH<br>3.2 m s^{-1} WS	University sports science male students (n=7).<br>Age 21 ± 1.5 y	Crossover, randomised	30 min self-paced cycling TT	Pre-cooling	900 mL cold beverage ingestion (2°C, 45 s) 35 min before exercise	**↑2.9% TD (p=0.03)**<br>**↑5.4% P (p=0.03)**
Choo et al., 2019 (122)	33.9 ± 0.2°C<br>42.5 ± 3.9 % RH	Recreationally active males (n=11).<br>Age 30 ± 6 y.<br>VO_2peak 51.1 ± 8.2 mL·kg $^{-1}$·min^{-1}	Crossover, randomised	15-RPE fixed 60 min cycling	Pre-cooling	7.5 g kg $^{-1}$ ice slushy ingestion (0.7± 0.1°C) 30 min before exercise	↓0.76% MPO (p>0.999)<br>↓1.27 % TWO (p>0.999)
De Carvalho et al., 2015 (109)	35°C<br>60% RH<br>0.5 m s^{-1} WS	Well-trained male athletes (n=10).<br>Age 25 ± 1 y.<br>VO_2max 67.2 ± 1.8 mL·kg $^{-1}$·min^{-1}	Crossover, counterbalance, randomised	Self-paced 40-km cycling time-trial	Per-cooling	Ad libitum cold water ingestion (10°C)	↓1.5% TT (p=0.425)
Ihsan et al., 2010 (96)	30°C<br>75 % RH	Endurance male trained subjects (n=7).<br>Age 27.7 ± 3.1 y	Crossover, counterbalance, randomised	40 km cycling TT	Pre-cooling	6.8 g kg^{-1} crushed ice ingestion (1.4±1.1°C) 30 min before exercise	↑6.9% MPO (p=0.06)<br>**↓6.5% TT (p=0.049)**
Lee and Shirreffs 2007 (118)	25.4 ± 0.4°C<br>60 ± 4% RH	Non-heat-acclimatized males (n=9).<br>Age 26 ± 6 y.<br>VO_2peak 50.0 ± 5.3 mL·kg $^{-1}$·min^{-1}	Crossover, randomized	90 min cycling at 50% VO_2peak + 95% VO_2peak TTE	Per-cooling	250 mL cold beverage ingestion (10°C) within each 2.5 min between the 30 and 40-min mark of the 90 min cycling	↔TTE (p=0.562)

Table 1. (continued)

Study	Ambient conditions	Participant characteristics	Design	Exercise protocol	Cooling timing	Cooling technique	Performance outcomes
Lee et al., 2008 (140)	AT<br>25.4°C<br>WBT<br>19.8°C<br>60% RH	Non-heat acclimated male (n=8).<br>Age 27 ± 4 y.<br>VO_2peak 53.8± 6.2 mL·kg^{-1}·min^{-1}	Crossover, counterbalance, randomised	90 min cycling at 50% VO_2peak	Per-cooling	400 mL cold beverage ingestion (10°C) at 30,45,60 and 75 min of exercise	↓3.75% EC (p=0.963)
Lee et al., 2008 (107)	35.0 ± 0.2°C<br>60.0 ± 1.0 % RH	Non-heat acclimated male (n=8).<br>Age 22 ± 4 y.	Crossover, counterbalance, randomised	Cycling at 65% VO_2peak TTE	Pre-cooling<br>Per-cooling	300 mL cold beverage ingestion (4°C) 30 min before and every 10 min during exercise	**↑11.9% TTE (p<0.01)**
Mundel et al.,2006 (112)	33.9 ± 0.2°C<br>27.9 ± 0.7 % RH	Healthy non-acclimated males (n=8).<br>Age 26 ± 7 y.<br>VO_2max 54 ± 5 mL·kg^{-1}·min^{-1}	Crossover, randomised	TTE at 65% W_{max}	Per-cooling	Ad libitum cold beverage ingestion (3.6±0.2°C)	**↑12.7% TTE (p=0.04)**
Riera et al, 2016 (124)	29 ± 0.7 °C<br>80 ± 0.02 % RH	Heat-acclimated trained male cyclists and triathletes (n=9).<br>Age 41 ± 17 y.<br>VO_2max 59 ± 11 mL·kg^{-1}·min^{-1}	Crossover, randomised	30 km cycling TT	Pre-cooling	7 g kg^{-1} cold beverage ingestion (3°C) 30 min before exercise	↓2% TT (p>0.05)

Table 1. (continued)

Study	Ambient conditions	Participant characteristics	Design	Exercise protoco	Cooling timing	Cooling technique	Performance outcomes
Saldaris et al., 2019 (125)	34.2 ± 0.9°C<br>52.9 ± 8.1% RH	Well-trained endurance male athletes (n=9).<br>Age 24 ± 4 y	Crossover, randomised	800 kJ cycling time trial	Pre-cooling	7.0 g kg^{-1} crushed ice ingestion (-0.3°C) 30 min before exercise	**↓7.2% TT ($p=0.012$)**<br>**↑7.8% MPO ($p=0.002$)**
Siegel et al.,2011 (113)	34.1 ± 0.1°C<br>49.5 ± 3.6% RH	Healthy males (n=10).<br>Age 24 ± 3 y.<br>VO_2peak 49.8 ± 4.7 $mL \cdot kg^{-1} \cdot min^{-1}$	Crossover, randomised	Running to exhaustion (treadmill) at VT1	Per-cooling	1.25 g kg^{-1} ice slurry ingestion (-1°C)	↔ RTE ($p=0.530$)
Siegel et al., 2012 (114)	34.0 ± 0.1°C<br>52 ± 3 % RH	Healthy non-heat acclimated male (n=8).<br>Age 26 ± 4 y.<br>VO_2peak 54.2 ± 2.5 $mL \cdot kg^{-1} \cdot min^{-1}$	Crossover, randomised	Running to exhaustion (treadmill) at VT1	Pre-cooling	7.5 g kg^{-1} ice slurry ingestion (-1°C) 30 min before exercise	**↑12.8% TTE ($p<0.05$)**
Stanley et al., 2010 (126)	33.7 ± 0.8°C<br>60.3 ± 2.0% RH	Trained male cyclists and triathletes (n=10).<br>Age 30 ± 5 y.<br>VO_2peak 60.0 ± 7.7 $mL \cdot kg^{-1} \cdot min^{-1}$	Crossover, counterbalance, randomised	Cycling TT (75% PPO x 30 min)	Pre-cooling	1 L ice slurry ingestion (-0.8± 0.1°C, 5 min) before the start of the time trial	↓1.9% TT ($p=0.263$)

Table 1. (continued)

Study	Ambient conditions	Participant characteristics	Design	Exercise protocol	Cooling timing	Cooling technique	Performance outcomes
Stevens et al., 2013 (127)	32-34°C<br>20-30% RH	Well-trained male triathletes (n=9).<br>Age 29.1 ± 3.6 y.<br>VO_2max 61.7 ± 4.7 mL·kg $^{-1}$·min^{-1}	Crossover, counterbalance, randomised	Triathlon (Olympic distance)	Per-cooling	10 g kg $^{-1}$ ice slurry ingestion (< 1°C) in the 17-45 min period of the cycle leg	**↓2.5% T (running phase) (p=0.03)**
Xu et al, 2021 (132)	38.1 ± 0.6°C<br>55.3 ± 0.3% RH	Non-acclimated male college athletes (n=7).<br>Age 20.3 ± 1.3 y.<br>VO_2max 60.7 ± 4.1 mL·kg $^{-1}$·min^{-1}	Crossover, randomised	TTE at 80% VO_2max	Pre-cooling	2.3 g ml kg^{-1} cold beverage ingestion (4°C) 30 min before exercise	↑4% TTE (p=0.493)
Yeo et al, 2012 (104)	27.6±0.2°C<br>28.4 ± 0.9% RH	Physically active male (n=8) and female (n=4).<br>Age 23.0 ± 2.2 y.<br>VO_2peak 54.3 ± 8.7 mL·kg $^{-1}$·min^{-1}	Crossover, counterbalance, randomised	Outdoor 10-km running TT	Pre-cooling	8.0 g kg^{-1} ice slurry ingestion (-1.4°C) 30 min before exercise	**↓0.6% TT (p=0.023)**
Zimmermann et al., 2017 (98)	34.9 ± 0.3°C<br>49.8 ± 3.5% RH	Female cyclists and triathletes (n=10). Age 28 ± 6 y	Crossover, randomised	800 kJ cycling time trial	Pre-cooling	7.0 g kg^{-1} crushed ice ingestion (0.5°C) 30 min before exercise	↓2.2% TT (p>0.05)

Table 1. (continued)

Study	Ambient conditions	Participant characteristics	Design	Exercise protocol	Cooling timing	Cooling technique	Performance outcomes
Zimmermann et al., 2017 (115)	35.0 ± 0.3°C<br>50.2 ± 2.1% RH	Healthy active males (=10).<br>Age 23 ± 3 y.<br>VO_2peak 48.5 ± 3.6 mL·kg $^{-1}$·min^{-1}	Crossover, randomised	Cycling 60 min<br>55% VO_2peak	Pre-cooling	7.0 g kg^{-1} crushed ice ingestion 30 min before exercise	↑0.3% TW (p>0.05)
**Menthol and Ice/Cold Beverages**							
Crosby et al., 2022 (105)	33.0 ± 3.0°C<br>46.0 ± 5.0% RH	Non-heat-acclimated male (n= 6) and female (n= 5).<br>Age 25 ± 5 y	Crossover, counterbalance, randomised	3 Minute Aerobic Test	Pre-cooling	25 mL L-menthol solution mouth rinse (0.1%, 5s) 1 min before the start of the test or<br>25 mL cold water mouth rinse (4◦C, 5s) 1 min before the start of the test	**MEN**<br>**↑13% RPO (p=0.05)a**<br>**↑6% RPO (p=0.05)c**<br>COLD<br>↔ RPO (p>0.05)a

Table 1. (continued)

Study	Ambient conditions	Participant characteristics	Design	Exercise protocol	Cooling timing	Cooling technique	Performance outcomes
Jeffries et al., 2018 (116)	35 ± 0.2°C<br>40 ± 0.5% RH	Non-heat-acclimated male (n=10).<br>Age 33 ± 9 y.<br>VO_2peak 52.4 ± 5.3 $mL \cdot kg^{-1} \cdot min^{-1}$	Crossover, randomised, single-blind	TTE at 70 %W_{max}	Per-cooling	1.25 g kg^{-1} ice slurry ingestion (10 s, 0.3±0.3°C) at 85% of baseline TTE<br>or<br>25 mL L-menthol solution mouth rinse (0.01%, 5 s, 19.5±0.5°C) at 85% of baseline TTE	**MEN**<br>**↑6% TTE (p=0.036)[a]**<br>↔TTE (p>0.05)[b]<br>**ICE**<br>**↑7% TTE (p=0.04)**
Riera et al., 2014 (108)	30.7 ± 0.8°C<br>78 ± 0.03 % RH	Heat-acclimated trained male cyclists and triathletes (n=12).<br>Age 42 ± 13 y.<br>VO_2max 59.9 ± 10.4 $mL \cdot kg^{-1} \cdot min^{-1}$	Crossover, randomised	20 km cycling TT	Pre-cooling<br>Per-cooling	190 mL ice (0.01%, 1°C ±0.7°C) or cold (0.01%, 3°C ±0.1°C) or thermoneutral (0.01%, 23°C ±0.1°C) menthol beverage ingestion before and after warm-up and every 5 km<br>or<br>same 3 beverages non-menthol flavoured before and after warm-up and every 5 km	**MEN**<br>**↓5.3% TT (p<0.02)**<br>**ICE**<br>**↓6.8% TT (p<0.02)**<br>**MEN + ICE**<br>**↓11% TT (p<0.03)**

Table 1. (continued)

Study	Ambient conditions	Participant characteristics	Design	Exercise protocol	Cooling timing	Cooling technique	Performance outcomes
Saldaris et al., 2020 (129)	35.3 ± 0.3°C<br>59.2 ± 2.5% RH	Long-distance male runners (n=12).<br>Age 25.3 ± 4.2 y.<br>VO_2peak 61.3 ± 4.3 mL·kg $^{-1}$·min^{-1}	Crossover, counterbalanc e, randomised	90 min run (3x 30 min on treadmill at 65% VO2peak) + TTF run at 100% VO2peak	Pre-cooling<br>Per-cooling	7.0 g kg^{-1} ice ingestion (-0.3 ± 0.3 °C) 30 min before exercise + 25 mL menthol solution mouth rinse (0.1%, 5s, 33.4± 0.5°C) each 15 min of TTF run (MIX)<br>or<br>7.0 kg^{-1} water ingestion (32.4 ± 0.9 °C) 30 min before + 25 mL menthol solution mouth rinse (0.1%, 5s, 33.4± 0.5°C) each 15 min and before TTF run (MEN)	**MIX**<br>**↑ 39.1% TTF (p=0.001)**<br>**MEN**<br>**↑34.4% TTF (p=0.02)**
Stevens et al.,2016 (130)	33°C<br>46% RH<br>4 m s^{-1} WS	Moderately trained heat-acclimated male runners (n=11).<br>Age 29 ± 9 y	Crossover, randomised	Self-paced 5-km running TT	Pre-cooling<br>Per-cooling	7.5 g kg^{-1} ice slurry ingestion (-1°C) 30 min before exercise<br>or<br>25 mL L-menthol solution mouth rinse (0.01%, 5s, 22°C) every 0.2 km mark of every 1 km	**MEN**<br>**↓2.7 % TT (p=0.01)[a]**<br>**↓3.8 % TT (p<0.01)[b]**<br>ICE<br>↔ TT (p=0.46)

Table 1. (continued)

Study	Ambient conditions	Participant characteristics	Design	Exercise protocol	Cooling timing	Cooling technique	Performance outcomes
Tran Trong et al., 2015 (131)	DBT<br>32.5±1.2°C<br>57%±0.05% RH<br>25.9 ± 0.7 km h^{-1} WS	Heat-acclimated trained male cyclists and triathletes (n=10).<br>Age 41 ± 17 y.<br>VO_2max 59 ± 11 mL·kg $^{-1}$·min^{-1}	Crossover, randomised	Outdoor 5x 4 km cycling + 1.5 km running TT	Pre-cooling<br>Per-cooling	190 mL cold beverage menthol-flavoured ingestion (0.025%, 3.1°C±0.6°C) in the warm-up and during the running segment of each block<br>or<br>190 mL ice-slurry menthol-flavoured ingestion (0.025%, 0.17°C±0.07°C) in the warm-up and during the running segment of each block	**ICE**<br>**↓6.2 TT (p<0.04)[a]**<br>**↓3.3 TT (p<0.002)[c]**

↔ no change, ↑ increase, ↓ decrease, AT: ambient temperature, DBT: dry-bulb temperature, EC: exercise capacity, FI: fixed intensity, kJ: kilojoules, kg: kilograms, km: kilometers, m: meters, mL: milliliters, min: minutes, MEN: menthol, MP: mean power, MPO: mean power output, P: power, P_{max}: maximal power output, PO: power output, PP: peak power, PPO: peak power output, RCP: respiratory compensation point, RH: relative humidity, RT: rate of fatigue, RPE: rate of perceived exertion, RPO: relative power output; RTE: run to exhaustion, s: seconds, SS: steady state; T: time, TD: total distance covered, TT: time trial, TTE: time to exhaustion, TTF: time to fatigue, TW: total work, VO_2max: maximal oxygen uptake, VO_2peak: oxygen uptake during peak exercise, VT1: first ventilatory threshold, W: work, WBGT: wet-bulb globe temperature, WBT: wet bulb temperature, WD: total work done, W_{max}: maximal aerobic power output, WS: wind speed and y:years.

[p] significance level; [a] vs. control; [b] vs. ice, [c] vs. cold beverage.
